# Reverse Genetic Approaches for the Generation of Recombinant Zika Virus

**DOI:** 10.3390/v10110597

**Published:** 2018-10-31

**Authors:** Ginés Ávila-Pérez, Aitor Nogales, Verónica Martín, Fernando Almazán, Luis Martínez-Sobrido

**Affiliations:** 1 Department of Microbiology and Immunology, University of Rochester Medical Center, 601 Elmwood Avenue, Rochester, NY 14642, USA; Gines_Perez@urmc.rochester.edu (G.A.-P.); aitor_nogales@urmc.rochester.edu (A.N.); 2 Department of Molecular and Cell Biology, Centro Nacional de Biotecnología (CNB-CSIC), Campus Universidad Autónoma de Madrid, 3 Darwin street, 28049 Madrid, Spain; veronica.martin@inia.es

**Keywords:** flavivirus, Zika virus (ZIKV), reverse genetics, infectious clone, full-length molecular clone, bacterial artificial chromosome, replicon, infectious RNA

## Abstract

Zika virus (ZIKV) is an emergent mosquito-borne member of the *Flaviviridae* family that was responsible for a recent epidemic in the Americas. ZIKV has been associated with severe clinical complications, including neurological disorder such as Guillain-Barré syndrome in adults and severe fetal abnormalities and microcephaly in newborn infants. Given the significance of these clinical manifestations, the development of tools and reagents to study the pathogenesis of ZIKV and to develop new therapeutic options are urgently needed. In this respect, the implementation of reverse genetic techniques has allowed the direct manipulation of the viral genome to generate recombinant (r)ZIKVs, which have provided investigators with powerful systems to answer important questions about the biology of ZIKV, including virus-host interactions, the mechanism of transmission and pathogenesis or the function of viral proteins. In this review, we will summarize the different reverse genetic strategies that have been implemented, to date, for the generation of rZIKVs and the applications of these platforms for the development of replicon systems or reporter-expressing viruses.

## 1. Introduction

### 1.1. Importance of Zika Virus in Human Health

Zika virus (ZIKV) is a recently emerged mosquito-borne virus, which in 2016 was declared as an international public health emergency by the World Health Organization (WHO, http://www.who.int/csr/en/) [1]. ZIKV is a member of the Flavivirus genus that belongs to the *Flaviviridae* family and is closely related to other mosquitoes-transmitted flaviviruses of public health relevance such as Dengue virus (DENV), Yellow fever virus (YFV), Japanese encephalitis virus (JEV) and West Nile virus (WNV) [2,3]. ZIKV was first isolated in 1947 of a sentinel rhesus monkey in the Zika forest of Uganda [4] and has been associated with sporadic human cases detected across Africa and Asia, resembling a mild version of DENV or Chikungunya virus (CHIKV) [5]. These similarities with DENV and CHIKV has interfered with ZIKV diagnosis and most probably underestimated the number of cases for ZIKV infections [6]. Symptomatic disease generally is present with a mild febrile illness characterized by fever, rash, muscle pain, headache and conjunctivitis, although as up to 80% of the ZIKV cases are asymptomatic [7,8,9]. However, the outbreak in the island of Yap in 2007 [10], French Polynesia in 2013–2014 [11,12] and the massive epidemic that emerge in Brazil in 2015 [13,14] have caused major concerns due to the association of ZIKV infection with severe congenital abnormalities, including microcephaly in infants and an increased risk of Guillain-Barré syndrome in adults [15,16,17,18]. ZIKV is mainly transmitted to people through the bite of an infected *Aedes* spp. mosquito (*Ae. Aegypti* and *Ae. Albopictus*) [19], which carries a high risk for pregnant woman due to the ability to cross the placenta and infected fetal nervous tissues [20]. In addition to maternal-fetal transmission, ZIKV can also be transmitted from mother to child during pregnancy or spread through sexual contact, breastfeeding, blood transfusion and non-human primate bites [21,22,23].

### 1.2. ZIKV Biology

**Figure 1:**
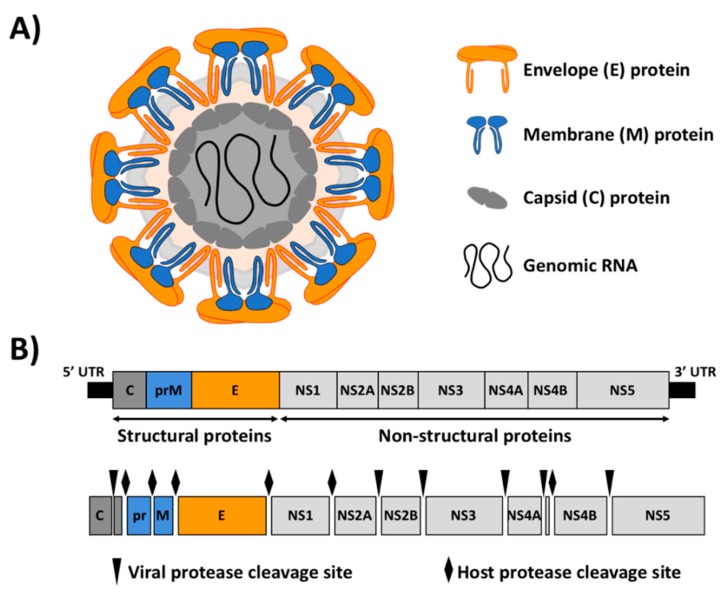
Zika virus (ZIKV) virion structure and genome organization. (**A**) Schematic representation of ZIKV virion structure: ZIKV virion surface is decorated with the E and M proteins, anchored in a lipid bilayer with an icosahedral-like symmetry. Under the viral lipid bilayer is the nucleocapsid composed of the vRNA genome associated with the C protein. (**B**) Genome organization and polyprotein processing: ZIKV genome (approximately 10.8 kb) is translated as a single polyprotein that is cleaved co- and post-translationally by viral (arrows) and host (diamonds) proteases to yield the three structural proteins C, M and E; and seven NS proteins NS1, NS2A, NS2B, NS3, NS4A, NS4B and NS5. The 5′ and 3′ untranslated regions (UTR) are indicated as black lines at the end of the viral genome.

ZIKV is an enveloped virus containing three structural proteins (Figure 1): the capsid (C) protein, the membrane (M) protein and the envelope (E) glycoprotein (Figure 1A). Inside the virion, the viral (v)RNA genome is complexed with multiple copies of the C protein, surrounded by the E and M proteins, which are anchored in a lipid membrane (Figure 1A). The surface of the mature virion has a icosahedral shell consisting of 90 E:M heterodimers (Figure 1A) [24]. The viral genome is made of a positive single-stranded RNA molecule of approximately 10.8 kb with a single open reading frame, flanked at the 5′- and 3′-ends by the viral untranslated regions (UTRs) (Figure 1B). ZIKV vRNA is translated by cap-dependent initiation producing a single polyprotein of approximately 3423 amino acids, which is co- and post-transcriptionally processed by both viral and cellular proteases into the three structural proteins (C, pre-Membrane (prM) and E) and seven non-structural (NS) proteins (NS1, NS2A, NS2B, NS3, NS4A, NS4B, NS5) (Figure 1B). The structural proteins are essential components of the virion and are involved in viral entry, fusion and assembly. The NS proteins are mainly involved in vRNA synthesis, assembly and regulation of the host cell responses [25]. Phylogenetic analysis of ZIKV have revealed two major genetic lineages (African and Asian), which have undergone substantial changes during the past 50 years. Sequence homology analysis indicated that the strains that have been responsible for the recent human outbreaks throughout the Pacific and the Americas are phylogenetically related to the most recent Asian lineage [26,27].

Like other flaviviruses, ZIKV entry occurs via attachment of the virions to the cell surface, a mechanism mediated by the viral E glycoprotein, followed by internalization by receptor-mediated endocytosis [28]. After virus internalization, the acid environment inside the endosome induces major conformational changes in the E glycoprotein that promote fusion of the viral and endosome membranes and the subsequent release of the viral genome into the cytoplasm of the infected cell. Once the genomic vRNA is released, it is translated to the viral polyprotein. As other positive-stranded RNA viruses, flaviviruses replication occurs on virus-induced host cell membranes, mainly characterized by invaginations of the endoplasmic reticulum (ER) membrane termed vesicle packets, which serve as scaffold for anchoring the viral replication complexes [29,30,31]. The immature viral particles are assembled within the ER, where the genomic vRNA is associated with the C protein and is packaged into ER-derived membranes containing the prM and E proteins. Virion maturation occurs in the *trans*-Golgi network during its transit through the cellular secretory pathway. Cellular furin protease mediate the cleavage of the prM, resulting in the release of the pr peptide and formation of mature virions containing the M protein that will be released from infected cells [32,33,34].

Because the very recent emergence of ZIKV, there are not currently approved vaccines or antivirals available to combat infection by this important human pathogen. Therefore, the generation of prophylactic (vaccines) and therapeutic (antivirals) options for the treatment of ZIKV are urgently required. The development of ZIKV reverse genetic systems provide investigators with a powerful tool for the development of vaccines and antivirals to counteract disease caused by this important human pathogen.

## 2. ZIKV Reverse Genetics

In 1981, Racaniello et al. reported, for the first time, the production of infectious virus by transfecting mammalian cells with a DNA plasmid containing the entire viral genome of poliovirus [35]. Since then, numerous reverse genetic technologies have been employed to recover recombinant viruses for the majority of viral families, including positive-sense RNA viruses such as coronavirus [36], picornavirus [37,38], flavivirus [39]; or negative-sense RNA viruses such as influenza [40] and arenavirus [41,42], among many others. Reverse genetic techniques have allowed investigators to generate recombinant viruses containing specific mutations to evaluate their contribution in viral replication and transcription, pathogenicity, virus-host interactions, inhibition of host cellular responses and host range or transmissibility [37]. Importantly, reverse genetic approaches have also been used for the developing of vaccines based on attenuated forms of the virus [37,40] and the generation of recombinant viruses harboring reporter genes to easily track viral infection, which have been resourceful to identify antivirals, host factors or for the in vitro and in vivo study of viral infections [43,44]. Viral reverse genetic approaches allow to recover infectious recombinant viruses upon transfection of a single (non-segmented viruses) or multiple (segmented viruses) DNAs into susceptible cells [40].

**Figure 2:**
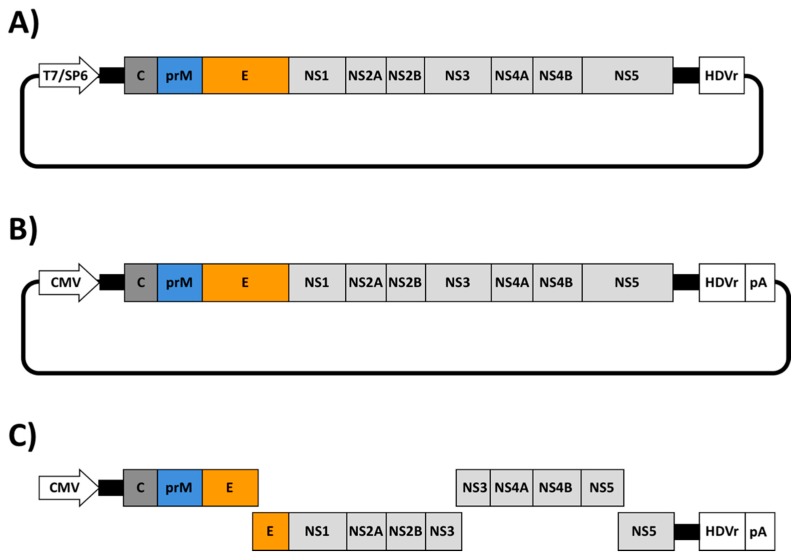
Schematic representation of ZIKV reverse genetic approaches. (**A**) Infectious RNA transcripts from full-length cDNA clones: A full-length genomic cDNA clone containing the ZIKV genome flanked by a prokaryotic promoter (e.g., T7 or SP6) and the hepatitis delta virus ribozyme (HDVr) is usually assembled in a low-copy plasmid. In this approach, in vitro transcription is required to produce viral RNA that is transfected into susceptible cells to initiate viral replication and transcription in the cytoplasm of transfected cells. (**B**) Full-length infectious genomic cDNA clones: A full-length infectious genomic cDNA clone containing the viral genome flanked by a polymerase II-driven promoter from cytomegalovirus (CMV) and the HDVr followed by a polymerase II terminator and polyadenylation signal (pA) is assembled in a low-copy plasmid. In this case, the full-length cDNA clone is transcribed in the nucleus of transfected cells by the cellular RNA polymerase II. Primary transcripts are translocated to the cytoplasm where further amplifications steps are conducted by the viral polymerase. (**C**) Infectious Subgenomic Amplicons (ISA): The ISA approach can be used for the production of infectious viruses from genomic DNA material, including pre-existing infectious cDNA clones, viral RNA or *de novo* synthesized DNA genomic sequences. The entire viral genome is amplified by overlapping PCR reactions with each PCR product containing 30–40 base pairs overlapping regions [45]. The first and last PCR products are flanked by the CMV promoter and the HDVr followed by a polymerase II terminator and pA signal, respectively. Co-transfected cDNAs result in self-assembly in the cytoplasm of susceptible cells and virus production.

Similar to other viruses, the recent emergence of ZIKV as an important human pathogen, has promoted the rapid development of numerous reverse genetic approaches to increase our knowledge of the molecular biology and pathogenesis of ZIKV. To date, three major reverse genetic strategies have been described to generate rZIKVs: (i) infectious RNA transcripts from a full-length complementary (c)DNA copy; (ii) DNA plasmids that are directly transfected to produce infectious ZIKV; and (iii) Infectious Subgenomic Amplicons (ISA) (Figure 2 and Table 1). The first strategy involves the in vitro transcription of an infectious RNA genome from a full-length cDNA copy containing the ZIKV genome under the control of a prokaryotic RNA polymerase promoter (e.g., T7 or SP6) (Figure 2A). In this case, the last nucleotide of the viral genome is followed by the hepatitis delta virus ribozyme (HDVr) sequence to produce synthetic RNAs bearing an accurate 3′ end (Figure 2A). Once transcribed, the vRNA is transfected into susceptible cells to recover infectious rZIKV. The second approach consist in the construction of a full-length infectious cDNA clone containing the viral genome flanked by a eukaryotic polymerase II-driven promoter at the 5′ end, mainly the cytomegalovirus (CMV) promoter and the HDVr sequence followed by a polymerase II terminator and polyadenylation signal (pA) at the 3′ end of the viral genome (Figure 2B). The full-length cDNA is usually assembled in a low-copy plasmid for its stable propagation in bacteria. In this system, the full-length infectious cDNA clone is directly transfected into susceptible cells where the vRNA is primarily transcribed in the nucleus by the cellular RNA polymerase II with further amplification steps in the cytoplasm driven by the viral polymerase. The last approach is based on the generation of overlapping double-stranded (ds)DNA fragments, covering the entire genome of ZIKV. In this case, the 5′ end fragment contains a polymerase II driven promoter (e.g., CMV) and the 3′ end fragment a HDVr sequence followed by a polymerase II terminator and polyadenylation signal (Figure 2C). This ISA approach involves the co-transfection of all the cDNA fragments into susceptible cells followed by the self-assembly of a full-length cDNA copy inside of the transfected cells by homologous recombination.

The construction of full-length cDNA clones for ZIKV has been hampered due to the toxicity of the viral genome, similar to other flavivirus, which is known to be toxic and unstable during its propagation in bacteria using standard plasmids [62,63,64,65]. This is attributed to the leaky expression of toxic viral proteins from cryptic prokaryotic promoters within the E and the NS1 coding sequences [66]. Therefore, several approaches have been developed to overcome the genome instability of flavivirus cDNA clones, including ZIKV. Those methods are summarized in Table 2 and described in detail in the following sections.

### 2.1. Infectious RNA Transcripts from Full-Length ZIKV cDNAs

In the last two years, several approaches based on the use of infectious RNA transcripts from full-length ZIKV cDNAs have been developed to overcome the toxicity problems associated with several sequences of ZIKV genome when amplified in bacteria. Those methods involve the use of low-copy number plasmids [46,47,48,49,50,51], the incorporation of an intron in the genome to disrupt toxic regions [42], in vitro ligation of cDNA fragments [52,53] and Gibson assembly [54,55], amount others.

#### 2.1.1. Construction of Full-Length ZIKV cDNA Clones Using Low-Copy Number Plasmids

A widely employed approach to overcome the toxicity of ZIKV and facilitate the stability of full-length cDNA clones consist in the use of low-copy number plasmids in which the cryptic prokaryotic promoters are maintained at a low level of expression, along with the use of prokaryotic transcription terminators to prevent the spurious transcription of foreign DNA sequences in bacterial host cells [63] (Table 2). Following this strategy, Shan-June et al. in 2016 [46] engineered the first ZIKV reverse genetic approach for the ZIKV FSS13025 strain that was isolated in 2010 from a 3-year-old patient from Cambodia [67]. In this approach, five RT-PCR fragments spanning the complete viral genome were individually cloned and assembled into a low-copy number plasmid using unique restriction sites present in the viral genome. Authors chose the low-copy number plasmid pACYC177 (15 copies of plasmid per cell) because it was previously used with other flavivirus cDNA clones [68,69]. Importantly, during the cloning procedure, the fragment spanning the viral prM-E-NS1 genes was reported to be toxic using a high-copy number plasmid. The T7 promoter and the HDVr sequence were engineered at the 5′ and 3′ ends of the ZIKV cDNA for in vitro transcription and the generation of the authentic 5′ and 3′ ends of the viral RNA, respectively. Using this approach, a rZIKV was successfully recovery after transfection of the infectious in vitro transcribed RNA in mammalian Vero cells. However, in vitro analysis showed that the rZIKV replicated at lower efficiency than the natural ZIKV isolate in both mammalian (Vero) and mosquito (C6/36) cells. Moreover, whereas the rZIKV produced plaques with homogenous morphology in Vero cells, the parental viral strain formed plaques with heterogeneous sizes. This phenotype correlated with what was observed in vivo using interferon receptor deficient (IFNAR−/−) A129 mice, where rZIKV was less virulent that the parental ZIKV.

In 2017, Mutso et al. [48] constructed a reverse genetic system based on the ZIKV Brazilian BeH81915 strain using a single low-copy plasmid. In this case, the bacterial artificial chromosome (BAC) plasmid pCC1-BAC was used to assemble the ZIKV genome under the control of a bacterial SP6 promoter. Notably, the rescued rZIKV showed similar in vitro grow kinetics in mammalian Vero cells, human brain endothelial cells (hCMEC/D3) or human choriocarcinoma placental epithelial (BeWo) cells than the natural ZIKV strain PRVABC59 isolated in Puerto Rico in 2015 [70].

Similarly, Nannamaliai et al. in 2017 [49] described the construction of a full-length cDNA clone of the historical 1947 Uganda ZIKV strain MR766 using a low-copy number plasmid. To that end, four RT-PCR fragments spanning the complete ZIKV MR766 genome were assembled in the plasmid pBR322 (around 20 copies per cell). However, the fragment containing the NS1 coding region was systematically instable during its propagation in bacteria, introducing deletion of 1 or 2 nucleotides. To bypass this problem, they cloned the toxic fragment in the linear vector pJAZZ-OC [71], which was used to assemble the full-length cDNA clone. pJAZZ-OC plasmid is derived from the linear dsDNA genome of coliphage N15 and can be maintained with 2–4 copies/cells [71] in the bacteria host. This plasmid has prokaryotic transcription terminators flanking the cloning site to eliminate the transcription into and out of the insert, which increases the stability of the insert in the bacterial host. Viral RNA transcripts were produced in vitro using the T7 RNA polymerase and infectious virus was obtained from RNA-transfected Vero cells. Importantly, the rZIKV MR766 showed similar grow kinetics in vitro in mammalian Vero or mosquito C6/36 cells than the parental MR766 ZIKV strain. Moreover, rZIKV MR766 recapitulated the pathogenic properties in vivo of the parental MR766 virus (IFNAR−/− A129 mice).

#### 2.1.2. Stabilization of Full-Length ZIKV cDNA by Mutational Inactivation of Cryptic *E. coli* Promoters (CEPs)

An alternative approach to reduce the toxicity related with the expression of CEPs consist in the inactivation of these sequences by the introduction of punctual silent mutations in the viral genome (Table 2). This approach was previously described to stabilize the full-length cDNA clones of JEV and DENV-2 [66]. Following this strategy, Münters et al. in 2018 [50] described the construction of full-length cDNA clones of the African 1947 Uganda MR766 and the Asian French Polynesia 2013 (H/PF/2013) strains of ZIKV. In this case, four fragments spanning the entire ZIKV genomes were assemble into the low-copy pFK plasmid [72] under the control of the phage T7 promoter using unique restriction sites. However, they consistently observed that the full-length cDNA clones were unstable during their propagation in bacteria. This problem was avoided with the introduction of punctual silent mutations to disrupt the CEPs present in the viral genome. Mutational inactivation of these cryptic promoters, which were predicted in silico to reside in the structural regions of MR766 and H/PF/2013 genomes, was sufficient to stabilize the full-length cDNA clones of both ZIKV strains. Furthermore, ZIKV cDNA clones were stable after five serial passages in *E. coli*. Both viruses were successfully rescue after transfection of Vero cells with RNA produced in vitro using the T7 RNA polymerase.

Recently, Zhao et al. in 2018 [51] also described the stabilization of a full-length cDNA clone from a ZIKV Brazilian isolated (SPH2015) by introducing silent mutations into the ZIKV genome to eliminate the activity of CEPs. However, one potential problem in this approach is that the introduction of multiple silent mutations can disrupt the viral RNA structure and affect viral replication and transcription, affecting the successful rescue or the phenotype of the recovered virus in cultured cells or in vivo (Table 2).

#### 2.1.3. Stabilization of Full-Length ZIKV cDNA Clones Using Intron Insertions

The introduction of short eukaryotic introns in the viral genome to disrupt toxic regions has been previously described to stabilize flavivirus cDNA clones [73]. Following this strategy, a full length cDNA clone of ZIKV GZ01, a virus strain isolated from a patient in China [74], was successful assembled by Liu Z-Y et al. [42] in 2017 to successfully rescue a rZIKV. Authors introduced a modified version of the group II self-splicing *P.li.LSUI2* intron [75,76] between the E and NS1 ZIKV coding regions to disrupt the toxic regions located in that region of the viral genome. The intronic sequences generally contain multiple stop codons, which interrupt the translation of the gene in bacteria (Table 2). The *P.li.LSUI2* intron, from the brown alga *Pylaiella littoralis* [77], was shown to have the ability to carry out efficient self-splicing under in vitro conditions [75]. Thus, authors used this *P.li.LSUI2* intron to produce vRNA transcripts with an intact ZIKV sequence. To construct the full-length cDNA clone, four RT-PCR fragments covering the entire full-length ZIKV genome were assembled under the control of the SP6 promoter in the low-copy plasmid pACNR1180 [78]. The intron sequence was chemically synthesized and cloned into the first fragment using overlapping PCR. Importantly, the intron-encoded protein sequence, required for self-splicing, was removed to prevent the splicing of the ZIKV cDNA in bacteria cells. In addition, the exon binding sequences of the inserted intron were modified to recognize the flanking ZIKV sequences in order to ensure the correct splicing. Using this approach, a rZIKV GZ01 was successfully recovered after the transfection of the in vitro-spliced RNA in BHK-21 cells (baby hamster kidney cell line). The recovered rZIKV GZ01 and the parental virus had similar grown kinetics and plaque morphology in vitro in both mammalian BHK-21 and mosquito C6/36 cells. Moreover, both viruses caused similar levels of neurovirulence in neonatal BALB/c mice, demonstrating that this approach can be a feasible option for rescue rZIKV [42].

#### 2.1.4. Construction of Full-Length ZIKV cDNA Clones Using In Vitro Ligation

A common strategy to overcome the instability associated with the construction of full-length cDNA clones of some flaviviruses has been the transfection of infectious vRNA transcripts from a full-length cDNA template, which is produced by in vitro ligation [79]. This method involved the partition of the viral genome in multiples fragments flanked by natural or engineered specific restriction sites that allows the systematic and precise assembly of a full-length cDNA by in vitro ligation. Following this method, Widman et al. [52] described in 2017 the successfully rescue of representative strains of ZIKV from the African (MR766) and Asian (H/PF/2013) lineages. In addition, they were able to rescue two rZIKV from two strains (SPH2015 and BEH819015) isolated in Brazil [52]. Authors generated a quadripartite system to disrupt the toxic ZIKV genomic regions by cloning the genome in four stable plasmids. Furthermore, natural nonpalindromic restriction endonuclease sites located near of toxic regions [52,80] were used to allow the directional ligation of the digested subgenomic fragments to generate a full-length cDNA clone. The resulting assembled product was then transcribed into RNA in vitro using the T7 polymerase. This method avoids the use of a bacterial host for the propagation of the full-length cDNA and therefore overcome the problems associated with cDNA instability (Table 2). Importantly, rZIKVs generated using this approach replicate in cultured cells similarly than their parental viral isolates. Moreover, rZIKVs were virulent in type I and type III interferon deficient AG129 mice, although slightly attenuated compared to their natural viral isolates [52].

Recently, the same reverse genetic approach was used by Gorman et al. in 2018 to rescue the African ZIKV strain Dakar 41525 [53]. Likewise, Deng et al. in 2017 also described the successful rescue of rZIKV SZ-WIV01, a ZIKV strain belonging to the Asian lineage [81]. In this case, authors combined the use of a quadripartite system with BglI overhang restrictions sites for in vitro ligation and assembly of the full-length cDNA clone.

#### 2.1.5. Construction of Full-Length ZIKV cDNA Clones Using Gibson Assembly

Gibson assembly is a molecular cloning method which allows the assembly of multiple overlapping DNA molecules in one reaction using a 5′ exonuclease, a DNA polymerase and a DNA ligase [82]. Like the in vitro ligation method described above, the entire viral genome is generated and maintained in multiples overlapping fragments to disrupt the toxic regions present in the viral genome (Table 2) [82]. Following this approach, in 2017, Weger-Lucarelli et al. developed a reverse genetic system for the ZIKV PRVABC59 strain [54]. In this manuscript, the viral genome of PRVABC59 was cloned in two separated pieces into the pACYC177 vector to disrupt the unstable NS1 region. To that end, the authors generated one vector with the T7 polymerase promoter sequence followed by the first third of the ZIKV genome. A second pACYC177 vector with the rest of the viral genome followed by the HDVr sequence was also generated. Both pACYC177 plasmids were engineered containing overlapping regions with unique restriction sites to allow reassemble of the viral genome. The full-length cDNA of ZIKV PRVABC59 was then generated by digestion and ligation using the Gibson assembly method. The resulting assembled product was then transcribed into RNA using the phage T7 polymerase and the product was transfected in Vero cells for the successful rescue of rZIKV PRVABC59. Like in the in vitro ligation approach described above, this method avoids the need to use a bacterial host for the propagation of the cDNA after the final assembly (Table 2). Importantly, the rZIKV PRVABC59 was able to replicate at similar levels to the natural virus isolate in both mammalian (Vero cells, BHK-21 cells, Huh7 human hepatoma cell line, JAR human placental cell line, amount others) or mosquito (C6/36 or Aag2) cells. Moreover, the levels of transmission in mosquitos and pathogenesis in type I and type III interferon deficient AG129 mice were also comparable between the genetically engineered and the natural ZIKVs isolates.

### 2.2. Full-Length Infectious ZIKV cDNA Clones

The use of reverse genetic approaches containing eukaryotic polymerase II-dependent promoters significantly simplifies the recovery of recombinant viruses as compared to the previously described procedures using phage T7 or SP6 RNA polymerase-based promoters, since direct plasmid transfection in susceptible cultured cells allows the rescue of recombinant viruses (Table 1). This approach involves the expression of the viral RNA in the nucleus of transfected cells from a polymerase II-dependent promoter with subsequent amplification steps in the cytoplasm by the viral polymerase. However, as described above, the main concern with this approach is the construction of full-length infectious ZIKV cDNA clones and the problems associated with stability of the viral genome during its propagation in bacteria. Several approaches have been developed to overcome the problems associated with genome instability of full-length infectious ZIKV cDNAs, including the use of intron sequences [57,58], assembly by circular polymerase extension cloning [59] or the use of BACs [48,56] (Table 2).

#### 2.2.1. Stabilization of Infectious Full-Length ZIKV cDNA Clones Using Introns

The first reverse genetic approach for the construction of a full-length infectious ZIKV cDNA clone was reported by Tsetsarkin et al. in 2016 [57]. Authors described the generation of an infectious cDNA clone for ZIKV Paraiba_01/2015, a virus isolated from serum of a febrile female subject in the Paraiba state in Brazil during the 2015 epidemic. To that end, four overlapping cDNA fragments spanning the entire ZIKV genome were individually cloned and assembled into the low-copy number plasmid pACNR1811 using conventional molecular cloning techniques. To restrict plasmid toxicity during the propagation of the cDNA in bacteria, they inserted two intron sequences in the NS1 and NS5 regions of the viral genome. Authors observed that the insertion of a single intron into the NS1 was sufficient for stable propagation of the infectious ZIKV cDNA clone but insertion of a second intron copy into the NS5 region was necessary to increase the plasmid yield in *E. coli.* The RNA polymerase II dependent CMV promoter and the HCVr sequences were engineered to flank the complete viral cDNA genome to ensure the successful rescue of the virus after plasmid transfection in Vero cells. Excision of the introns was carried out by the cellular machinery in the nucleus of transfected cells and, therefore, the resulting RNA was identical to the virus sequence and could initiate the replication/transcription steps in the cytoplasm (Table 2). The cDNA-derived rZIKV replicated efficiently in multiple cell lines, including those of placental and neuronal origin. Likewise, Schwarz et al. in 2016 also reported the construction of a plasmid carrying the complete genome of the prototype MR766 ZIKV African strain under the control of a CMV promoter [58]. In this case, the full-length cDNA clone was toxic during propagation in bacteria but incorporation of an intron in the NS1 region was sufficient to stabilize the cDNA clone.

#### 2.2.2. Assembly of Full-Length Infectious ZIKV cDNA Clones Using Circular Polymerase Extension Cloning (CPEC)

As an alternative to bypass the inherent problem associated with the toxicity of ZIKV cDNA genomes during the propagation in bacteria, Setoh et al. in 2017 used a CPEC reaction approach [83] to construct a full-length infectious cDNA clone of the Brazilian ZIKV strain Rio Grande do Natal (RGN) [59]. The approach is based on the assembling of multiple cDNA fragments in the right order using a polymerase extension mechanism (Table 2). Briefly, eight overlapping cDNA fragments covering the entire viral genome flanked by the CMV promoter (5′ end) and HDVr and pA signal (3′ end), were mixed and subjected to a CPEC reaction using the Q5 high fidelity DNA polymerase. During the denaturalization step associated with the PCR reaction, the overlapping ends of contiguous cDNA anneal to each other and the PCR reaction generates a circular end product. The CPEC products were then used to directly transfect Vero cells without any additional manipulations, allowing the rescue of the rZIKV. The rZIKV-RGN was able to replicate in Vero, human A459 and mosquito C6/36 cells. Due to the absence of an RGN natural isolated, the author compared the replication of the rZIKV-RGN with the Asian strain ZIKV MR766, showing that rZIKV-RGN replicated less efficiently than ZIKV MR766. Moreover, ZIKV-RGN was detected in serum of infected IFNAR−/− A129 mice after 4 -5 days, however the infection was asymptomatic with 100% of survival. In contrast, ZIKV-RGN infection of pregnant IFNAR−/− A129 dams showed severe fetal disorder.

#### 2.2.3. Construction of Full-Length Infectious ZIKV cDNA Clones Using BACs

Another strategy used to engineer infectious full-length cDNA clones of positive-strand RNA viruses is the use of BACs [62,84,85,86,87,88]. Following this strategy, we have recently developed an infectious cDNA clone of ZIKV RGN strain [56]. To that end, the full-length cDNA copy of the ZIKV RGN genome was assembled in the BAC plasmid pBeloBac11 [89], a synthetic low-copy number plasmid based on the *E. coli* F-Factor [90]. This plasmid allows the stable maintenance of large DNA fragments and minimizes the toxicity associated with the propagation of full-length viral genomes in bacteria (Table 2). The full-length copy of the viral genome was cloned using four overlapping synthesized individual cDNA fragments and assembled into the pBeloBac11 using conventional cloning methods and unique restriction sites present in the viral genome. In this case, the viral genome was flanked at the 5′ end by the CMV promoter and at the 3′ end by the HDVr followed by the bovine growth hormone (BGH) termination and polyadenylation sequence to produce synthetic RNAs bearing authentic 3′-ends of the viral genome. The ZIKV-RGN clone was highly stable during passage in *E. coli* and infectious virus was recovered after direct transfection of susceptible Vero cells. Grown kinetics and plaque morphology showed than the recover rZIKV-RGN replicated efficiently in Vero and human A549 cells, with a homogeneous plaque morphology. Importantly, IFNAR−/− A129 mice succumbed to infection with the rZIKV-RGN in a dose-dependent manner. As the manipulation of BAC cDNA clones is relatively easy, we also used our system to construct a full-length infectious rZIKV clone containing an amino acid change (A175V) in the viral NS2A protein. Notably, this single and conserved amino acid change impaired viral RNA synthesis and viral production in cultured cells and attenuated the virus in vivo.

Likewise, Mutso et al. [48] in 2017 used a similar strategy to construct an infectious full-length cDNA clone based on the Brazilian ZIKV strain BeH81915. To that end, four DNA fragments spanning the entire viral genome were assembled into the BAC plasmid pCC1-BAC. In this case, the second intron of the human beta globin gen was introduced in the capsid region to increase the stability of the cDNA clone.

### 2.3. Infectious Subgenomic Amplicons (ISA) for the Generation of rZIKVs

ISA reverse genetics were development a few years ago by Aubry et al. [91]. In this approach, simple transfection of overlapping dsDNA fragments, covering the entire genome of an RNA virus flanked at the 5′ end by a CMV promoter and at the 3′ end by the HDVr sequence and pA signals, were sufficient to rescue multiple flaviviruses, including DENV, JEV or WNV [91,92]. This approach is based on spontaneous recombination between the overlapping cDNA fragments that allows the assembly and synthesis of a full-length cDNA copy of the complete viral genome in transfected cells. This approach could facilitate the rescue of recombinant RNA viruses from cDNA without requiring propagation in bacteria or in vitro RNA transcription. Following this strategy, Gadea et al. described in 2016 the rescue of rZIKV MR766 African [45] and BeH819015 Asian [61] strains. In addition, Atieh et al. in 2016 used the ISA technology for the generation of two reverse genetic systems based on the genome of ZIKV H/PF/2013 and Dakar 1984, representative members of Asian and African ZIKV lineages, respectively [60]. However, a potential concern with this method could be associated to a low efficiency of recombination of the cDNA fragments into transfected cells. Therefore, the amount of virus produced after the transfection using the ISA approach could be low, negatively impacting the successful rescue of recombinant viruses harboring, for instance, mutations that affect one or several steps in the replication cycle of the virus.

## 3. Rescue of rZIKVs Using Reverse Genetics Approaches

The generation of rZIKVs using reverse genetic approaches is currently well established and although is used by multiple laboratories worldwide, it is possible to find multiple variations between these approaches among different research groups. Moreover, the origin of the ZIKV strain used and the reverse genetic approach selected might require introducing modifications and/or optimization procedures for the successful rescue of rZIKVs.

**Figure 3:**
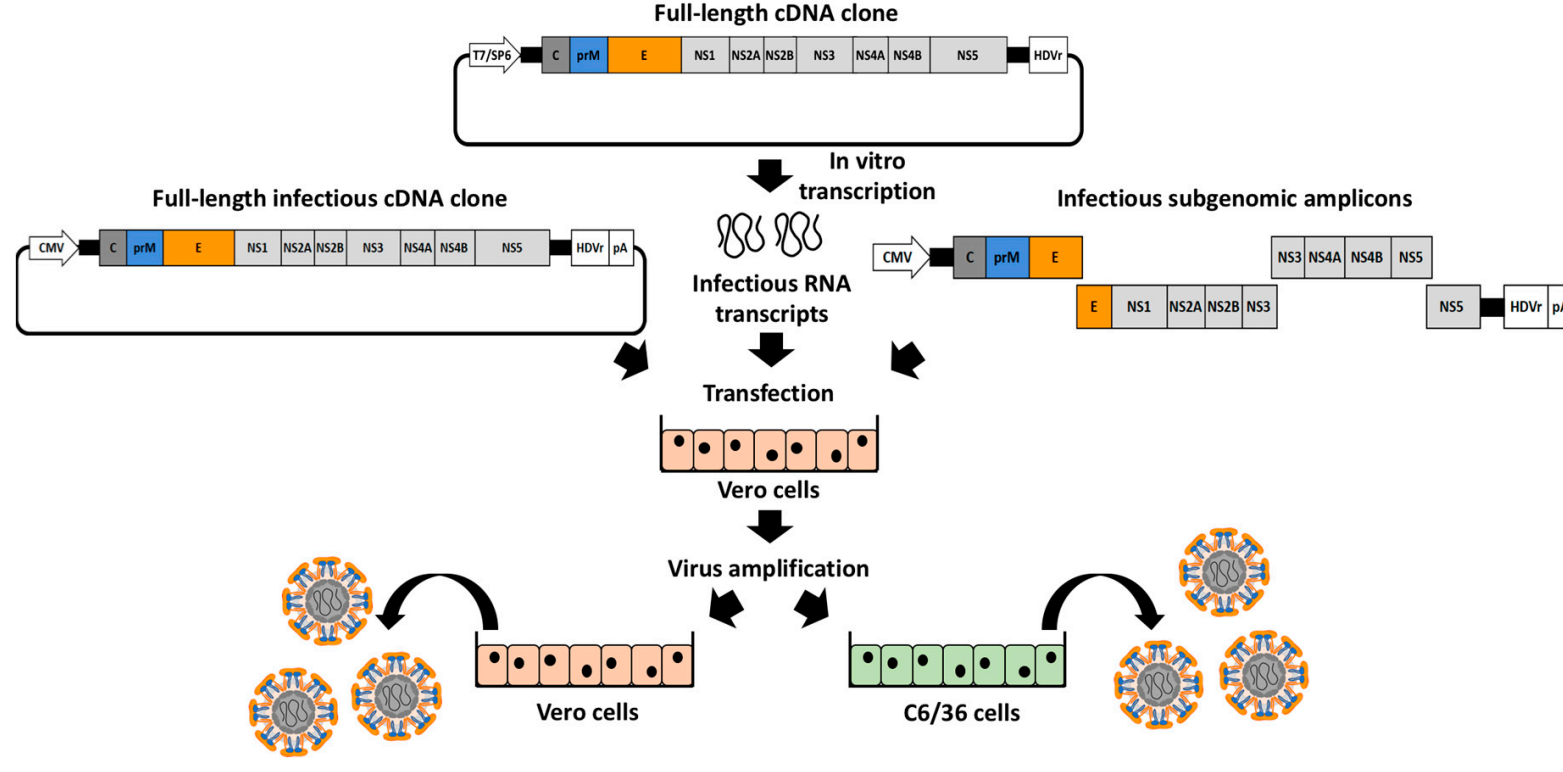
Schematic representation for the generation of rZIKV: Mammalian Vero cells are transiently transfected with infectious RNA transcribed in vitro from a full-length cDNA clone (top), full-length infectious cDNA clones (**left**) or infectious subgenomic amplicons (**right**) (see also Figure 2). After 3–4 days post-transfection, when cytopathic effect is observed, rZIKVs present in tissue-culture supernatants can be recover for titration or viral amplification in mammalian (Vero, **left**) or insect (C6/36, **right**) cells.

To generate rZIKVs (Figure 3), susceptible cells (commonly mammalian Vero cells) are transiently transfected with the genetic material encoding the viral genome, as infectious RNA transcribed in vitro from a full-length cDNA clone (Figure 2A), full-length infectious cDNA clones (Figure 2B) or using infectious subgenomic amplicons (Figure 2C). Like the viral RNAs, the infectious RNA transcripts are directly expressed into the cytoplasm producing the viral proteins required for viral replication and transcription. However, both full-length infectious clones and subgenomic amplicons require a primary transcription in the nucleus by the cellular RNA polymerase II and the successive translocation of the vRNA into the cytoplasm. In general, after transfection of either cDNA clones or infectious RNAs, a clear cytopathic effect is observed at 3-4 days post-transfection with viral titers ranging between 1×10^4^ and 1×10^7^ plaque forming units (PFU) per mL in the tissue culture supernatants of transfected cells. However, both time and titers will depend on the viral strain and the reverse genetic approach used to generate the rZIKVs. Moreover, after transfection, one or multiple steps of viral amplification in mammalian (e.g., Vero) or mosquito (e.g., C6/36) cells could be required to obtain a working stock with higher viral titers (Figure 3). The successful rescue of rZIKVs using reverse genetic approaches can be evaluated by performing classical plaque (PFU/mL) or immunofluorescence (immunofluorescent forming units, FFU/mL) assays.

## 4. Applications of ZIKV Reverse Genetic Approaches

### 4.1. ZIKV Replicons

Subgenomic replicons systems are powerful tools to study viral replication in the absence of virus entry or virion assembly, since they contain all the elements needed to produce effective viral replication in susceptive host cells but lack one or multiple viral structural genes [36,72,93,94,95]. Due to the lack of viral structural genes, these replicon systems are non-infectious, allowing the study of viral replication without biosafety concerns associated with the work of infectious viruses [85,96]. Thus, replicons represent a safe tool to study, among others, viral replication, transcription or the subcellular localization of the viral replication complexes. Moreover, replicons systems have been used for the identification of compounds with antiviral activity or host factors involved in viral replication using high-throughput screening settings [97,98,99,100].

**Figure 4:**
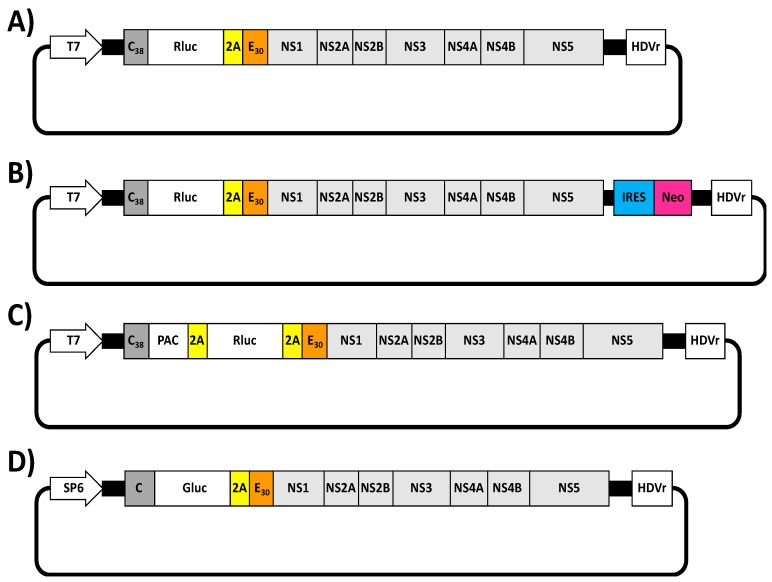
ZIKV replicons. (**A**,**B**) Schematic diagram of Rluc-expressing ZIKV replicons: ZIKV replicons expressing Rluc constructed by Xie et al. [101] are shown. In these replicons, ZIKV structural proteins were replaced by the Rluc reporter gene followed by the FMDV 2A protease sequence (2A, yellow). This cassette was flanking by the N-terminal 38 amino acids of C protein (C_38_, dark gray) and the last 30 amino acids of E protein (E_30_, orange) fused in-frame with the viral downstream NS proteins (**A**). A stable reporter ZIKV Rluc-expressing replicon was constructed inserting the encephalomyocarditis virus internal ribosomal site (IRES, blue) followed by a neomycin resistance gene (Neo, pink) into the first 28 nucleotides of 3′ UTR (**B**). (**C**) Schematic representation of a PAC- and Rluc-expressing ZIKV replicon: The ZIKV replicon expressing a PAC and a Rluc genes separated by the FMDV 2A protease sequence is shown [105]. Both foreign viral genes, PAC and Rluc, followed by the FMDV 2A protease sequence were introduced between of the first N-terminal 38 amino acids of C protein (C_38_, dark gray) and the last 30 amino acids of E protein (E_30_, orange) in-frame with the viral NS1 protein. (**D**) Schematic diagram of a Gluc-expressing ZIKV replicon: The ZIKV replicon expressing the Gluc reporter gene constructed by Mutso et al. is indicated [48]. The Gluc reporter gene followed by the FMDV 2A protease sequence was introduced downstream of the C protein (dark gray) and the last N-terminal 30 amino acids of E protein (E_30_, orange) fused in-frame with the NS1 ZIKV protein. T7 and SP6: prokaryotic T7 or SP6 promoters. HDVr: Hepatitis delta virus ribozyme sequence.

The first ZIKV replicon was described by Xie et al. in 2016 [101] (Figure 4A). The replicon was constructed using the infectious cDNA clone of ZIKV FSS13025 strain by replacing the viral structural genes with the Renilla luciferase (Rluc) reporter gene, followed by the foot-and-mouse disease virus (FMDV) 2A protease sequence to allow the cleavage of Rluc from the viral polyprotein. Importantly, the Rluc-2A cassette was flanked by the first 38 amino acids of the viral C protein (C_38_) that contain the flavivirus-conserved cyclization sequence required for viral RNA replication [102,103] and the last 30 amino acids of E protein (E_30_) fused in-frame with the downstream NS1 protein (Figure 4A). The E_30_ region was maintained to ensure proper translocation of the NS1 protein into the lumen of the ER [103]. To evaluate viral replication, in vitro RNA transcripts synthesized by the T7 RNA polymerase were electroporated into Huh-7 cells and Rluc signal was evaluated. As a proof of concept and to demonstrate the susceptibility of this replicon approach to identify compounds with anti-ZIKV activity, the authors showed that Rluc signal was suppressed using the broad antiviral NITD-008 in a dose-dependent manner [104]. Moreover and to obtain a Huh-7 stable cell line expressing the viral replicon, they engineered a second ZIKV replicon containing Rluc and the Neomycin (Neo) resistance gene (Figure 4B). The Neo gene driven by an encephalomyocarditis virus internal ribosomal entry site (IRES) was introduced downstream of the first 28 nucleotides of 3′ UTR. Importantly, authors demonstrated that the generated Neo resistant stable cell line maintains the ZIKV replicon for several passages and expressed high levels of Rluc.

An alternative strategy was performed by Li et al. in 2017 [105] (Figure 4C). Authors generated a bicistronic ZIKV replicon of SZ-WIV001 strain [81], in which the selection gene puromycin *N*-acetyl-transferase (PAC) and the reporter Rluc were separated by a FMDV 2A protease sequence (Figure 4C). In this case, the PAC-2A-Rluc construct was followed by a second 2A protease sequence and the cassette was introduced, in frame, between the C_38_ and the E_30_ sequences (C_38_-PAC-2A-Rluc-2A-E_30_) (Figure 4C). Murine BHK-21 stable cell lines constitutively expressing the viral replicon were produced after transfection of the ZIKV RNA replicon produced in vitro (T7) and selection with puromycin. In addition, the replicon was stably maintained in BHK-21 cells during multiple passages. Importantly, authors were able to adapt this technology to evaluate ZIKV inhibitors using a high throughput screening assay.

Recently, in 2018, Münters et al. described the construction of a set of Rluc ZIKV replicons using the full-length cDNA clones of ZIKV Asian H/PF/2013 or African MR766 strains, which contained silent mutation to disrupt the CEPs [50]. The viral structural proteins were replaced by the Rluc-2A cassette flanked by the first 34 amino acids of ZIKV C protein (C_34_) and the last 24 amino acids of the E protein (E_24_). Authors found that Rluc signal correlated with the amount of viral NS proteins. Moreover, the replicon induced morphological changes resembling ZIKV infection, highlighting the potential of this approach to study some steps of ZIKV infection.

Lastly, a ZIKV replicon expressing the Gaussian luciferase (Gluc) reporter gene was designed by Mutso et al. in 2017 using the full-length cDNA clone of the Brazilian ZIKV strain BeH819015 (Figure 4D) [48]. In this case, the Gluc reporter gene was inserted followed by the FMVD 2A protease sequence between the C protein and the last 30 amino acids of the E protein (E_30_) fused in-frame with the NS1 protein. The activity of the replicon was demonstrated in mammalian (BHK-21 and Vero) and mosquito (C6/36) cells.

### 4.2. Replicating Competent, Reporter Gene-Expressing rZIKVs

**Figure 5:**
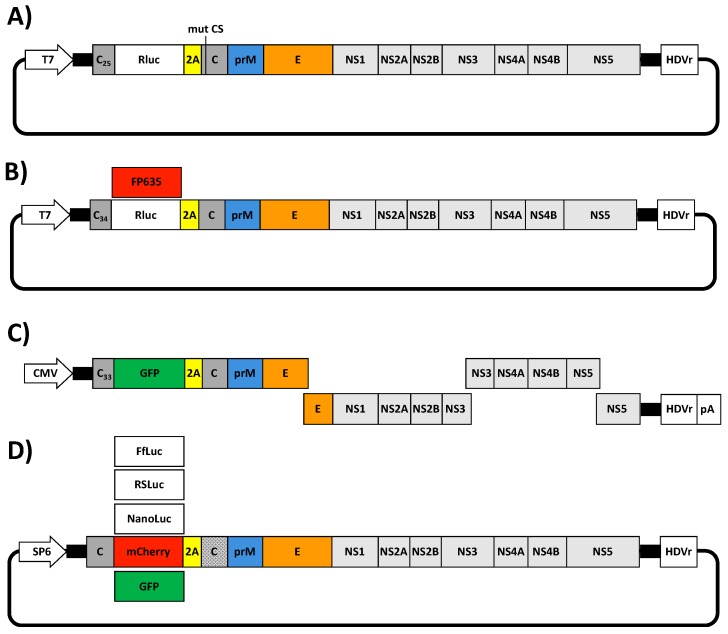
Reporter gene-expressing rZIKVs. (**A**) Schematic diagram of a Rluc-expressing ZIKV cDNA clone: A full-length cDNA clone expressing the Rluc reporter gene (white box) was constructed by Shan et al. [46]. Rluc was introduced downstream of the first 25 amino acids of the C protein (C_25_, dark gray) followed by the FMDV 2A protease sequence (yellow box) fused in-frame with the viral ORF. Silent mutations changing the flavivirus-cyclization sequence were introduced in the full-length C protein (mut CS). (**B**) Schematic representation of a full-length ZIKV cDNA expressing Rluc or FP635: Full-length cDNA clones expressing Rluc (white box) or FP635 (red box) were generated by Münters et al. [50]. Reporter genes were introduced downstream the first 34 amino acid of the C protein (C_34_, dark gray) followed by the FMDV 2A protease sequence in-frame with the viral downstream proteins. (**C**) Schematic diagram of a GFP-expressing ZIKV generated by ISA: A ZIKV cDNA clone expressing GFP was constructed by Gadea et al. [45] using the ISA approach. The GFP was introduced downstream of the first 33 amino acid of the C protein (C_33_, dark gray) followed by the FMDV 2A protease sequence in-frame with the viral ORF. (**D**) Representation of full-length ZIKV cDNAs expressing luciferase and fluorescent proteins: Full-length ZIKV cDNA clones expressing NanoLuc, FfLuc, RSLuc (white boxes), GFP (green box) or mCherry (red box) were constructed by Mutso et al. [48]. Reporter genes were individually introduced downstream of the complete C protein followed by the FMDV 2A protease sequence fused in-frame with the downstream viral ORF. The codon sequence of the downstream copy of the viral C protein was altered to reduce potential recombination (dotted gray). T7 and SP6: prokaryotic T7 or SP6 promoters. CMV: cytomegalovirus promoter. HDVr: Hepatitis delta virus ribozyme sequence. pA: simian virus 40 late polyadenylation signal.

The development of reverse genetic approaches has provided investigators with the possibility of generating replication-competent viruses expressing fluorescent or luminescent reporter genes [106]. The stable incorporation of foreign reporter genes in viruses have allowed the effective tracking of viral infection in vitro and in vivo without the need of laborious secondary approaches to identify the presence of the virus [43,44,106]. Moreover, recombinant reporter-expressing viruses have the advantage that they can be used in high throughput screenings assays for multiple applications, including the identification of compounds with antiviral activity [107,108], host factor involved in viral infection [109,110] and the presence of neutralizing antibodies [111,112,113,114], among others. To date, several rZIKVs expressing reporter genes have been described [45,46,48,50]. In all cases, independently of the reverse genetic approach used to generate the reporter gene expressing rZIKV, a similar strategy was used, consisting in the introduction of the reporter genes followed by the FMDV 2A protease sequence upstream of the viral open reading frame. In addition, a copy of the C protein or part of the its N-terminal region that contains the flavivirus-conserved cyclization sequence [102,103] was introduced upstream of the reporter gene to allow successful viral replication (Figure 5).

Shan et al. in 2016 engineered a full-length cDNA ZIKV FSS13025 infectious clone expressing Rluc (rZIKV-Rluc) (Figure 5A) [46]. In this system, Rluc was introduced downstream of the first 25 amino acid of the C protein (C_25_) followed by the FMDV 2A protease sequence and in frame with the ZIKV downstream proteins (Figure 5A). Importantly, silent mutations were introduced into the original C protein in the flavivirus-cyclization sequence (amino acids 14–17) to reduce the potential recombination between the parental and the duplicated C protein regions (Figure 5A). The rZIKV-Rluc was successful rescued after transient transfection in Vero cells of the full-length in vitro transcribed (T7) ZIKV RNA. However, the rZIKV-Rluc had a reduced plaque size phenotype compared with the parental rZIKV, indicating that insertion of Rluc was deleterious for the virus.

Münters et al. in 2018, described the construction of two Rluc-expressing rZIKVs using the full-length cDNA clones of ZIKV MR766 and H/PF/2013 [50]. In this case, the Rluc-2A cassette was introduced downstream of the first 34 amino acids of the C protein (C_34_) and in frame with the ZIKV downstream proteins (Figure 5B). In this case, the duplicated region of the C protein was not altered. Expression of Rluc was confirmed for both rescued viruses. However, as in the previous case, rZIKV-Rluc (MR766 or H/PF/2013) replicated at lower extend as compare to non Rluc-expressing rZIKV MR766 or H/PF/2013, further suggesting that insertion of Rluc affected viral fitness. Notably, the reporter gene was unstable and lost after 3 passages. In this case, the rapid loss of the Rluc could be due to recombination events between the partial duplication of the capsid regions. To have a system suitable for microscopy, Münters et al. replaced the Rluc reporter gene by the coding sequence of turbo far-red fluorescent protein FP635, a mutant version of the red fluorescent protein from the sea anemone *Entacmaea quadricolor* [115]. In this case, a SV40 nuclear localization sequence was introduced in frame with the FP635 protein for nuclear export of the fluorescent protein and to avoid interference with viral replication events in the cytoplasm of infected cells. Although, authors did not evaluate the replication properties of these viruses, they demonstrated that FP635-expressing rZIKVs were suitable for live cell imaging.

Using the ISA method, Gadea et al. described in 2016 the generation of a rZIKV MR766 expressing the green fluorescent protein (GFP) reporter gene (rZIKV-GFP) [45] (Figure 5C). GFP was introduced downstream of the first 33 amino acids of C protein (C_33_) followed by the FMDV 2A protease sequence in-frame with the viral ORF (Figure 5C) [45]. The rZIKV-GFP was successfully rescued after electroporation of several fragments covering the complete genome under the control of the CMV promoter. However, rZIKV-GFP titers were ~1.5 log lower compared with the rZIKV without GFP. Likewise, the plaque phenotype of the rZIKV-GFP was smaller than rZIKV without GFP, indicating that insertion of GFP resulted in viral attenuation. Moreover, rZIKV-GFP was unstable and the virus lost GFP expression after a limited number of passages on Vero cells.

More recently, Mutso et al. described the construction of several reporter-expressing rZIKVs using a BAC system and a SP6 promoter (Figure 5D) [48]. They inserted the sequences of the foreign reporter genes fused to the FMDV 2A protease sequence between two copies of C protein, altering the codon usage of the downstream copy to reduce potential recombination events (Figure 5D). Using this strategy, they generated rZIKVs expressing Firefly luciferase (FlLuc), red shift luciferase from *Luciola italica* (RSLuc) [116], NanoLuc, mCherry or GFP (Figure 5D). Moreover, they inserted a ubiquitin sequence between the FMDV 2A protease sequence and the viral polyprotein in order to help the proteolytic cleavage. However, inclusion of the ubiquitin sequence after the 2A protease sequence was irrelevant for viral rescue and stability. Although the replication of these reporter-expressing rZIKVs were not directly compared with either a rZIKV or a natural isolated, as previously described for the other reporter-expressing rZIKVs, limited stability of the rZIKVs expressing reporter genes was observed, except for NanoLuc which remained stable during four serial passages in cultured cells. These and previous results highlight the important concerns related to the instability of reporter-expressing rZIKVs. Further investigation, including new approaches for the expression of reporter genes in the viral genome are necessary to generate stable rZIKV expressing reporter genes for their use as valid viral surrogates to study ZIKV infection in cultured cells or in validated animal models of infection.

## 5. Conclusions

The development of ZIKV reverse genetic systems has provided researchers with powerful methods to study multiple aspects of the biology and pathogenesis of ZIKV in vitro and in vivo. For instance, the ability to manipulate the genome of ZIKV to generate recombinant viruses with specific mutations have allowed investigators to gain a detailed understanding of the ZIKV-host interactions and associated disease [49,56,57]. Moreover, reverse genetic approaches have been essential to develop novel and more effective strategies to prevent and control ZIKV infections [117,118,119]. Likewise, generation of ZIKV replicons or replicating-competent rZIKV expressing reporter genes represent and excellent approach for the identification of compounds with anti-viral activity for the treatment of ZIKV infection [45,46,101,105]. In this review, we have discussed the three major strategies that have been reporter for the generation of rZIKVs: i) generation of infectious RNA transcripts from a full-length cDNA copy; ii) full-length infectious genomic cDNA clones; and, iii) Infectious Subgenomic Amplicons (ISA) (Figure 2 and Table 1). Like other flavivirus, ZIKV cDNA sequences in bacterial has been hindered by the presence of cryptic bacterial promoter in the viral genome. To overcome the problems associated with the instability of ZIKV cDNA clones, several approaches based on the use of low-copy number plasmids, BACs, intron-insertion, in vitro ligation of cDNA fragments and in vitro assembly by Gibson method or by CPEC have been used. Although all those approaches have their advantages and disadvantages (Table 2), they have been not compared side by side. Therefore, it cannot be possible to determine which systems are better for the genetic stability and successful generation of rZIKV. Moreover, successful rescue of rZIKVs could strongly dependent of several biological factors such as the viral strain selected and technical factors such as transfection methods. Finally, the generation of rZIKVs expressing reporter genes, together with the development of ZIKV replicons, constitute important tools for the identification of antiviral drugs against this important human pathogen that are compatible with the use of high throughput screenings settings. However, to date, there are some concerns regarding the stability of currently available reporter-expressing rZIKVs, which will require and guarantee further investigation.

## Figures and Tables

**Table 1:** ZIKV reverse genetics techniques.

Approaches	Advantages	Disadvantages	Ref.
Infectious RNA transcripts from full-length ZIKV cDNAs	○ vRNAs are directly electroporated into cells ○ RNA electroporation is generally more efficient than transfection	○ Need an in vitro RNA transcription step ○ Genome instability of flavivirus cDNA clones in bacteria ○ Error rate of RNA in vitro transcription can produce undesired mutation	[42,46,47,48,49,50,51,52,53,54,55]
Full-length infectious ZIKV cDNA clones	○ Plasmid containing the viral genome is directly transfected into the cells ○ In vitro transcription is not required	○ Genome instability of flavivirus cDNA clones in bacteria	[48,56,57,58,59]
Infectious subgenomic amplicons (ISA)	○ Rapid and easy construction ○ cDNA assembly occurs into the cells	○ Nonhomogeneous populations by error in the recombination events	[45,60,61]

**Table 2:** Strategies to avoid toxicity of full-length constructs.

Approaches	Advantages	Disadvantages	Ref.
Low-copy number plasmid	Cryptic promoters are maintained at low level of expression	Low plasmid yield Flavivirus genome are often unstable	[46,47,48,49]
Bacterial artificial chromosome (BAC)	Minimization of toxicity by a strictly controlled replication leading to only one plasmid per cell. Stable maintenance of large DNA fragments	Low plasmid yield Manipulation of big DNA constructs	[48,56]
Inactivation of cryptic *E. coli* promoters (CEP)	CPEs are inactivated	Introduction of punctual mutation can disrupt the viral RNA structure and viral fitness	[50,51]
Intron insertion	Expression of toxic regions is interrupted in bacteria	Introduction of external sequences in the viral genome	[42,57,58]
In vitro ligation	Non-required propagation of full-length cDNA in bacteria	Viral genome is maintained in multiple fragments in bacteria Low ligation efficiency Low virus recovery efficiency	[52,53]
Gibson assembly or Circular polymerase extension cloning (CPEC)	Non-required propagation of full-length cDNA in bacteria Rapid assembly in one step	Viral genome is maintained in multiple fragments in bacteria Low virus recovery efficiency Error rate of the reaction can produce undesired mutations	[54,55,59]
